# Cost-Effectiveness of Tucatinib in Human Epidermal Growth Factor Receptor 2–Positive Metastatic Breast Cancer From the US and Chinese Perspectives

**DOI:** 10.3389/fonc.2020.01336

**Published:** 2020-08-04

**Authors:** Qiuji Wu, Weiting Liao, Mengxi Zhang, Jiaxing Huang, Pengfei Zhang, Qiu Li

**Affiliations:** ^1^Department of Medical Oncology, Cancer Center, West China Hospital, Sichuan University, Chengdu, China; ^2^West China Biomedical Big Data Center, Sichuan University, Chengdu, China

**Keywords:** tucatinib, metastatic breast cancer, HER2-positive, cost-effectiveness, Markov model

## Abstract

**Background:** The clinical evaluation of HER2CLIMB trial showed a 21. 9-month median overall survival with the triplet regimens of tucatinib, capecitabine, and trastuzumab (TXT) for patients with human epidermal growth factor receptor 2 (HER2) –overexpressing metastatic breast cancer. From the payer's perspective of the United States and China, a cost-effectiveness analysis was conducted to evaluate the costs and benefits of adding tucatinib in this study.

**Methods:** We constructed a Markov model for the economic evaluation of adding tucatinib to trastuzumab plus capecitabine in patients with HER-2 positive metastatic breast cancer in the United States and China. The model was conducted with a 10-year time horizon, and the health status was divided into three states: progression-free survival, progressing disease, and death. The health utility scores were consistent with published literature with similar patient status. The transition probabilities were derived from the survival data of the HER2CLIMB study. The unit prices of medicines were obtained from the West China Hospital, Red Book, and published literature. Outcomes were measured in quality-adjusted life-years (QALYs), and incremental cost-effectiveness ratio, which robustness was evaluated by deterministic and probabilistic sensitivity analyses.

**Results:** Compared with the two-drug regimen of trastuzumab plus capecitabine (TX), the addition of tucatinib increased 0.21 QALY, with an increasing cost of $146,995.05 and $19,022.97 in the United States and China, respectively. The incremental cost-effectiveness ratios (ICERs) for the TXT versus TX was $699,976.43 in the U.S. and $90,585.57 in China, both of which are higher than their respective threshold of willingness to play. Deterministic sensitivity analysis shows that the price of tucatinib is the parameter that has the most significant impact on ICERs, but it does not change the results of the model. Probability sensitivity analysis shows that the probability of cost-effective for TXT is 0 in the base case.

**Conclusion:** In the United States and China, tucatinib combined with trastuzumab and capecitabine is not cost-effective for patients with HER-2 positive metastatic breast cancer.

## Introduction

Cancer statistics in 2020 show that the incidence of breast cancer ranks first among female tumors (276,480 cases) and the second-highest mortality rate (42,170 death) in the United States (1). In China, breast cancer is the most common cancer among women, with 268.6 thousand people diagnosed with breast cancer in 2015 (2). These cases in China account for 17.6% of all newly diagnosed breast cancers and 11.1% of all breast cancer deaths worldwide (2, 3). An estimated 5.7% of incidence cases of breast cancer cases are distributed in Stage IV or metastatic in the United States (4). In China, up to 21.4% of patients with breast cancer have distant metastases at the initial diagnosis (5). Even after undergoing surgery and standard treatment, 20–30% of patients will relapse within 10 years, of which two-thirds are distant metastases (6, 7).

Human epidermal growth factor receptor 2 (HER2), as an oncogene for tumorigenesis, is overexpressed in 20–25% of invasive breast cancers, closely related to invasion, metastasis, and prognosis (8–10). In the advent of trastuzumab, anti-HER2 therapies have led to a significant improvement in overall survival in early and advanced patients with HER2-positive breast cancer. However, most patients ultimately develop the progressive disease and die. Furthermore, up to 40–50% of HER2-positive breast cancer patients will develop brain metastases (11, 12). Better options for the prevention and treatment of brain metastases are needed. There is no standard treatment for patients with HER2-positive metastatic breast cancer (MBC) that have progressed after treatment with trastuzumab, pertuzumab, and trastuzumab emtansine (T-DM1). Treatment options at this time include lapatinib in combination with capecitabine, trastuzumab and other chemotherapy (such as vinorelbine or gemcitabine), or participation in a clinical trial. A substantial number of novel anti-HER2 treatments are being investigated extensively in the preclinical and clinical settings, including novel antibody-drug conjugates (such as trastuzumab deruxtecan), other small-molecule tyrosine kinase inhibitors (TKIs) (such as tucatinib, neratinib, and pyrotinib).

Tucatinib is an orally bioavailable, small-molecule tyrosine kinase inhibitor. Different from other small molecules TKIs, tucatinib can selectively inhibit HER2 (13). In June 2017, tucatinib received orphan drug designation from the US Food and Drug Administration (FDA) for treatment of breast cancer patients with brain metastases (14). Tucatinib, trastuzumab, and capecitabine (TXT) were shown to improve overall survival compared with trastuzumab and capecitabine in the HER2CLIMB (ClinicalTrials.gov identifier, NCT02614794.) clinical trial (21.9 vs. 17.4 months), besides also significantly extending progression-free survival (PFS) compared with trastuzumab and capecitabine (TX) (7.8 vs. 5.6 months) (15). Based on these data, the FDA approved tucatinib in combination with chemotherapy (trastuzumab and capecitabine) for patients with HER2-positive MBC who have received one or more prior treatments in April 2020 (16).

The implications of tucatinib in the treatment of HER2 positive MBC are considerable, given the significant potential population of patients eligible to receive the therapy. Once tucatinib is approved in more countries, the widespread use of the drug may substantially increase the costs of breast cancer care. Moreover, the global breast cancer burden in women is rising in countries regardless of income level. Under the influence of COVID-19, the world economy and health has changed in profound and almost universal ways, adding more pressure to the growing shortage of limited medical resources. Cost-effectiveness analysis as an important tool for assessing whether new therapies provide clinical benefits at a reasonable cost is increasingly recognized. In this study, we assessed the appropriate price range of tucatinib for Chinese and US payers through cost-effectiveness analysis and explored the economics of new drugs in developing and developed countries.

## Materials and Methods

### Patients and Intervention

Our model is to simulate the treatment of patients with HER2-positive metastatic or recurrent breast cancer in the HER2CLIMB trial. The hypothetical cohort included patients over 18 years of age who have received a treatment course of trastuzumab, pertuzumab, and trastuzumab emtansine (15). Eligible patients were randomly assigned in a 2:1 ratio to treatment with TXT or TX. Patients in the TXT arm were administered 300 mg tucatinib twice daily throughout the treatment period. Besides, patients in both groups received trastuzumab (8 mg/kg for initial therapy followed by 6 mg/kg for maintenance) on day 1 and capecitabine (1,000 mg/m2) twice daily on days 1–14 repeated every 3 weeks (15). Patients continued to receive the current treatment plan until unacceptable toxicity, disease progression, withdrawal of consent, or study closure (15).

### Model Construction

**Figure 1:**
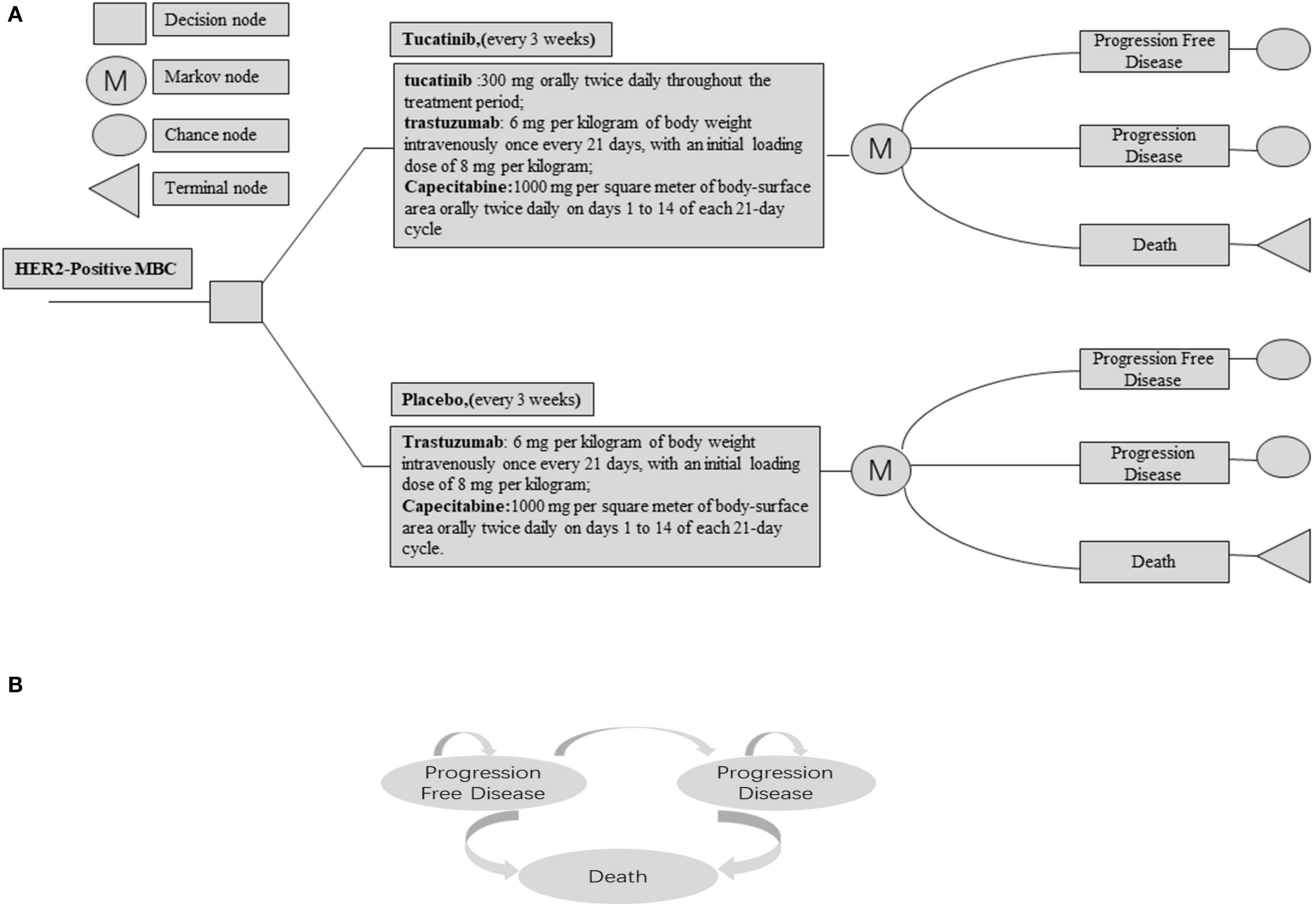
**(A)** Abbreviated decision tree and Markov model used to compare two strategies for treating patients with HER2-Positive Metastatic Breast Cancer. **(B)** The influence diagram shows a network of three health states linked by transitional variables.

We constructed a decision analysis Markov model through TreeAge Pro 2011 software (TreeAge, Williamstown, MA) to simulate the process of TXT or TX treatment for HER-2 positive MBC and to predict the 10-year costs and survival benefits of the two strategic therapies. A Markov decision process (MDP) is a stochastic model to a final decision process. Unlike the deterministic model, the Markov model results will change each time they are solved even though the initial conditions remain unchanged (17). The model consists of three mutually incompatible health states: progression-free survival (PFS), progression disease (PD), and death. The output of this model includes total cost, quality-adjusted life year (QALY), and incremental cost-effectiveness ratio (ICER). ICER is defined as the difference in costs between TXT and TX groups divided by the difference in effects. The ICER between the two groups was compared with the WTP threshold of $150,000 per QALY in the USA and $30,447.09 /QALY (three times GDP per capita) in China, respectively (18, 19). According to the HER2CLIMB trial, all patients in the model were randomly assigned to two groups with PFS status (Figure 1). The cycle length of the model is 3 weeks, which is consistent with the treatment cycle of the patients in the HER2CLIMB trial, and a half-cycle correction was applied (20).

**Figure 2:**
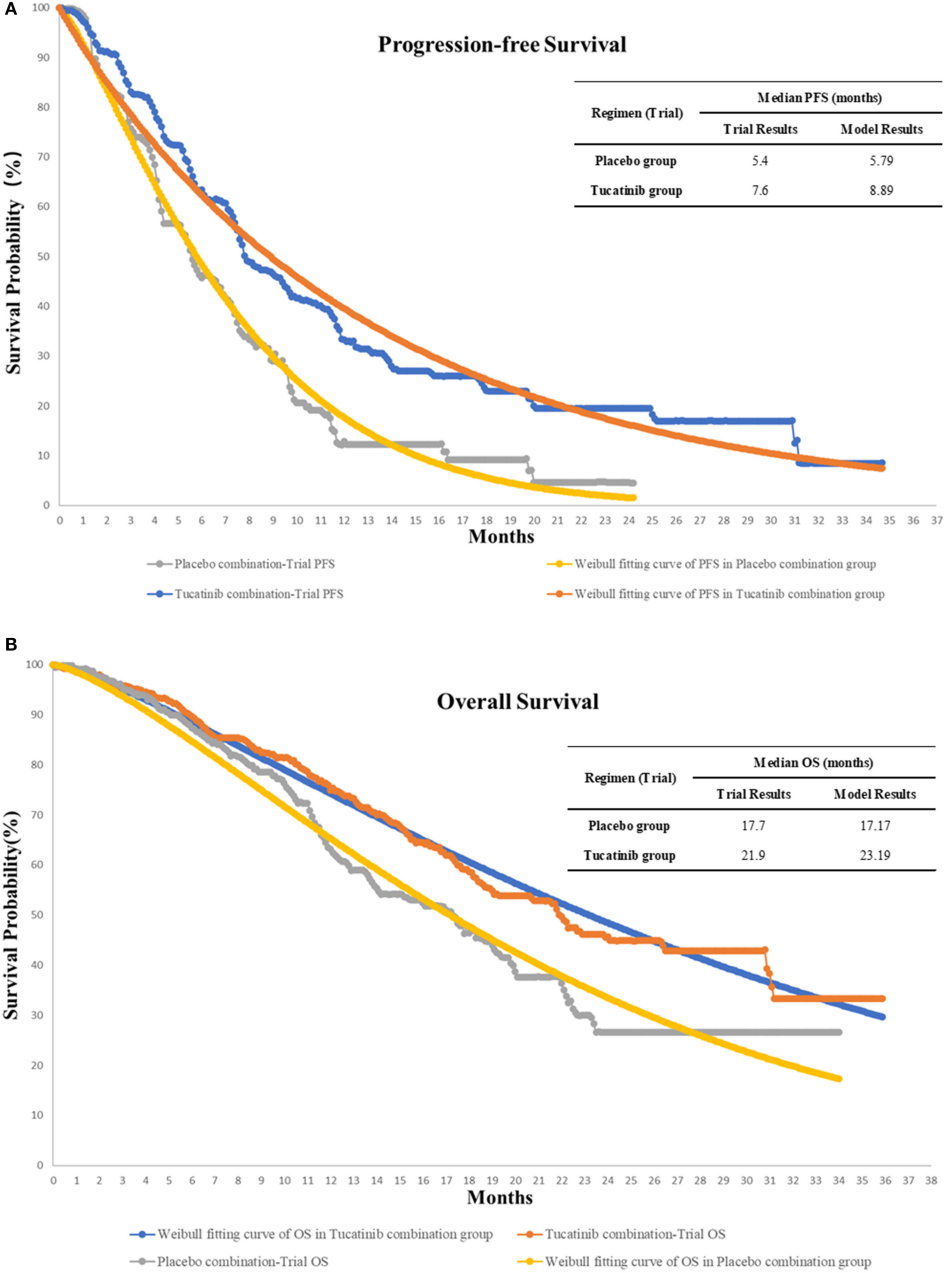
The original Kaplan-Meier PFS **(A)** and OS **(B)** curves from the HER2CLIMB trial, Weibull fitting curves, and the validation of our model of treatment strategies for HER2-Positive Metastatic Breast Cancer. OS, overall survival; PFS, progression-free survival.

The OS and PFS probabilities were extracted from the published OS and PFS curves of the HER2CLIMB trial by WebPlotDigitizer software (version 4.2; https://apps.automeris.io/wpd/index.zh_CN.html) (15), and these survival data were then used to fit parametric survival models using the algorithm derived by Hoyle et al. (21). The Weibull survival model results of the two groups are shown in Figure 2. Based on the fitted Weibull model, we can estimate the time-dependency transition probability from PFS to PD and PD to death in each cycle using the following formula: P(t → t + 1) = 1 − exp [λ(t)^γ^ − λ(t + 1) ^γ^)], where t stands for the current cycle number in the Markov model (22). The mortality rate in the PFS state for each age group was estimated based on Chinese and US life tables in the model (23, 24).

### Cost and Utilities

The cost of each group is assessed from the perspective of the Chinese and US payers, including the cost of tucatinib, trastuzumab, capecitabine, management of grade 3–4 adverse events (AEs), administration, best supportive care, and test. These costs come from Red Book, published literature, West China Hospital of Sichuan University, and Chinese national drug prices (25–37). The price of lapatinib in China was used in the base-case analysis because the tucatinib is not yet marketed. All expenses are listed in Table 1. We assumed the patients in both groups received best supportive care (BSC) after progression for absent treatment data in the sequence line. US and China costs associated with health care services were inflated to 2020 values according to the US and China consumer price index (38, 39). We converted all costs to US dollars [$1 = ¥6.9851 (February 2020)]. A discount rate of 3% per year was used for the costs and utility value involved in the model (40). The utility value represents the health-related quality of life for each state of health, ranging from 0 for death to 1 for perfect health. The utility values of this model were obtained from published literature with a health state similar to the HER2CLIMB trial (41–43).

**Table 1:** Model parameters and assumptions.

**Parameter**	**USA value**		**China value**		**Distribution**
	**Mean**	**Range**	**Mean**	**Range**	
Tucatinib per cycle, $	12,950 (22)	9,065–16,835	1,002.03	701.42–1,302.64	⋎
Trastuzumab per cycle, $	3,669.08 (22, 23)	2,568.36−4,769.80	952.01	666.41–1,237.61	Fix in PSA
Capecitabine per cycle, $	955.50 (22, 23)	668.85–1,242.15	189.60	132.72-246.48	Fix in PSA
BSC/cycle, $	2,933 (24)	2,053.1–3,812.9	807 (31)	564.9–1,049.1	⋎
Computed tomography imaging, per cycle, $	448 (25)	313.6–582.4	84.56	59.19–109.9	⋎
Cost of managing adverse events, per event, $					⋎
Palmar–plantar erythrodysesthesia	8.31 (26)	5.82–10.80	3.57 (26)	2.50–4.64	⋎
Diarrhea	1,183.7 (26)	828.59–1,538.81	12.79 (26)	8.95–16.63	⋎
ALT/AST increased	76 (27)		24.15 (32)	16.90–31.40	⋎
Fatigue	6,908 (28)	4,835.6?−8,980.4?	103.00 (32)	72.10–133.90	⋎
Anemia	13,679 (29)	9,575–17,783	921.10 (33)	348.60–1,494.32	⋎
Nausea, vomiting	5,246 (28)	3,672.2–6,819.8?	39.60 (34)	27.72–51.48	⋎
Stomatitis	10,073.67 (30)	7,051.57–13,095.77	42.20 (32)	29.54–54.86	⋎
Neutropenia	9,910 (29)	6,937–12,883	411.93 (32)	288.35–535.51	⋎
Utilities					⋎
PFS	0.86 (35–37)	0.602–1	0.86 (35–37)	0.602–1	β
PD	0.71 (35–37)	0.497–0.923	0.71 (35–37)	0.497–0.923	β
Discount rate, %	3 (0–5) (17)				β

### Sensitivity Analysis

A series of deterministic sensitivity analyses were conducted to explore the impact of uncertainty in our assumptions on treatment efficacy, utilities, and cost. Variables in a deterministic sensitivity analysis were varied in the range confidence interval or ± 30%. Besides, we investigated the possibility of TXT being cost-effective when the cost of tucatinib, utility value, and WTP threshold changed over a broader range. Based on the distribution characteristics of each parameter, gamma distributions were used for cost parameters, and the beta distributions were adopted for probability and health utility values. Then, we performed 1,000 Monte Carlo simulations to conduct probabilistic sensitivity analyses. The results of univariate sensitivity analyses were given as tornado diagram, and probabilistic sensitivity analyses were expressed as cost-effectiveness acceptability curves. We also analyzed the possibility of adding tucatinib being cost-effective in the brain metastasis subgroup.

## Results

### Base Case Results

Table 2 shows the results of the basic analysis. The model projected that the patients treated with TXT yielded 1.10 QALYs, which was 0.21 QALYs more than patients receiving TX. The use of tucatinib, capecitabine plus trastuzumab cost an additional $19,022.97, resulting in an ICER of $90,585.57 per QALY compared with capecitabine plus trastuzumab for patients with MBC in China. In the United States, the ICER was $699,976.43 per QALY.

**Table 2:** Summary of one- and multi-way deterministic and probabilistic sensitivity analyses.

**Assumption**	**Incremental** **cost ($)**	**Incremental** **benefit, QALY**	**ICER, per QALY ($)**	**Probability of** **cost-effectiveness (%)**
**CHINA**				
**Base case**				
WTP $30,447.09/QALY	19,022.97	0.21	90,585.57	0
WTP $80,000/QALY	19,022.97	0.21	90,585.57	21.9
WTP $100,000/QALY	19,022.97	0.21	90,585.57	74.5
**Subgroup**				
Brain metastases	18,049.06	0.32	56,403.31	0
**Utilities**				
PFS utility 1.0	19,022.97	0.25	76,091.88?	0
PD utility 1.0	19,022.97	0.20	95,114,85	0
PFS and PD utilities 1.0	19,022.97	0.23	87,708.57	0
**Cost**				
Tucatinib at 50% cost	14,078.15	0.21	67,038.81	0
Tucatinib at 10% cost	10,122.29	0.21	48,201.38	4.1
**USA**				
**Base case**				
WTP $150,000/QALY	146,995.05	0.21	699,976.43	0
WTP $500,000/QALY	146,995.05	0.21	699,976.43	0.1
WTP $800,000/QALY	146,995.05	0.21	699,976.43	80.3
**Subgroup**				
Brain metastases	129,429.57	0.32	404,467.41	0
**Utilities**				
Stable disease utility 1.0	146,995.05	0.25	587,980.20	0
Progressing utility 1.0	146,995.05	0.18	816,639.16	0
Stable and progressing utilities 1.0	146,995.05	0.23	639,108.91	0
**Cost**				
Tucatinib at 50% cost	84,851.03	0.21	404,052.52	0
Tucatinib at 10% cost	35,135.82	0.21	167,313.43	17.4

*ICER, incremental cost-effectiveness ratio; OS, overall survival; PFS, progression-free survival; PD, progression disease; QALY, quality-adjusted life-years; WTP, willingness to pay*.

### Subgroup Analysis

For patients with brain metastasis, TXT costs $18,049.06 more than TX with an additional 0.32 QALYs, resulting in an ICER of $56,403.31 per QALY in China. From the US perspective, the ICER was $404,467.41 per QALY.

### Sensitivity Analysis

**Figure 3:**
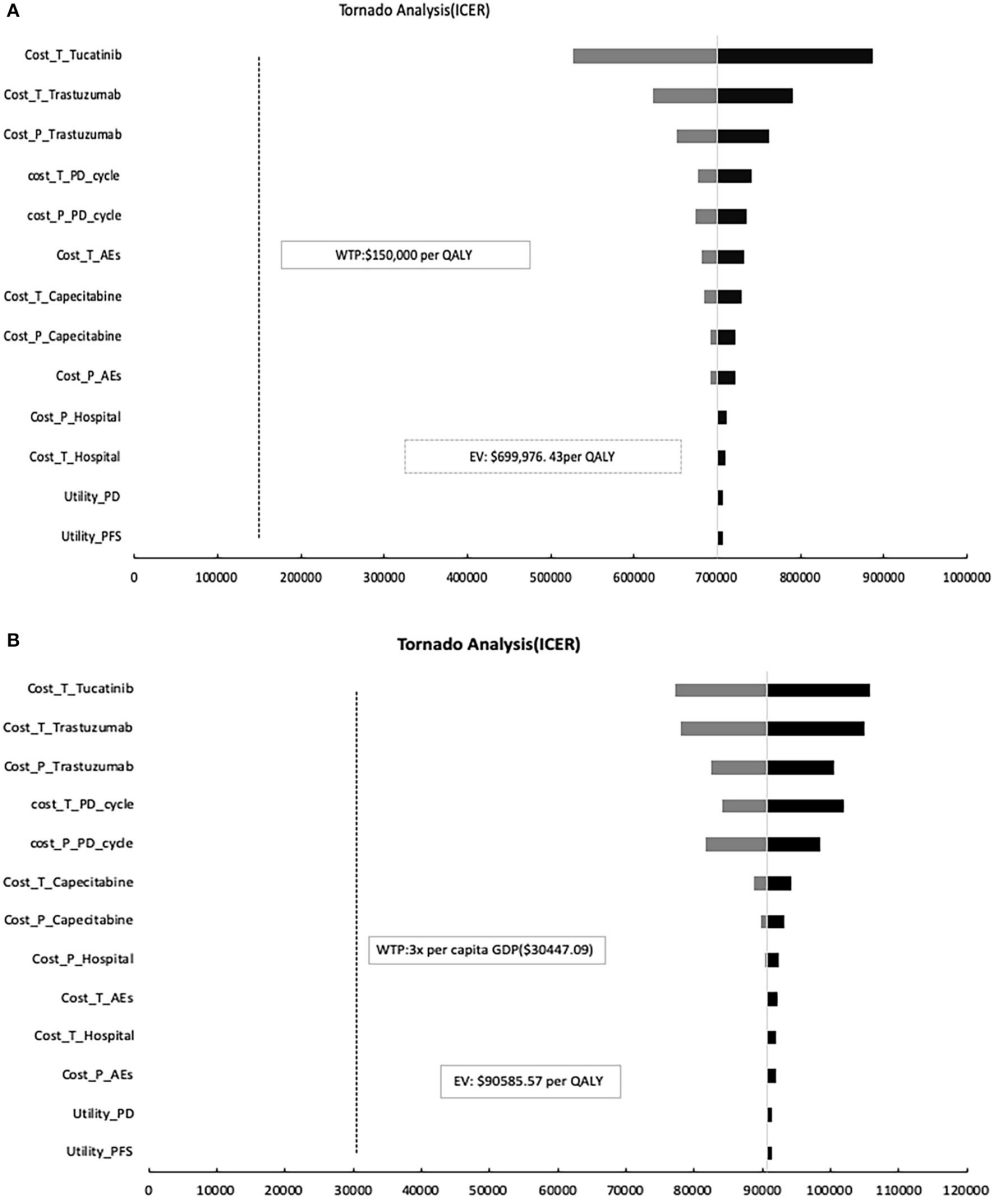
One-way sensitivity analysis. This diagram shows the incremental cost effectiveness ratio (ICER) of TXT vs. TX for different model input parameters in the United States **(A)** and China **(B)**, respectively. TXT, tucatinib, trastuzumab, and capecitabine; TX, trastuzumab plus capecitabine; PFS, progression-free survival; PD, progression disease; AEs, adverse events.

Figure 3 shows a tornado diagrams from a one-way sensitivity analysis. The most sensitive parameter is the price change of tucatinib in China and the United States, resulting in ICERs ranging from $77,189.02 to $105,715.57 and $527,420.51 to $885,926.80 per QALY, which are also higher than the WTP threshold set in the model. When perfect utilities were assigned to both stable and progressing disease states, the cost of TXT decreased to $87,708.57 per QALY gained and $639,108.91 per QALY, respectively.

**Figure 4:**
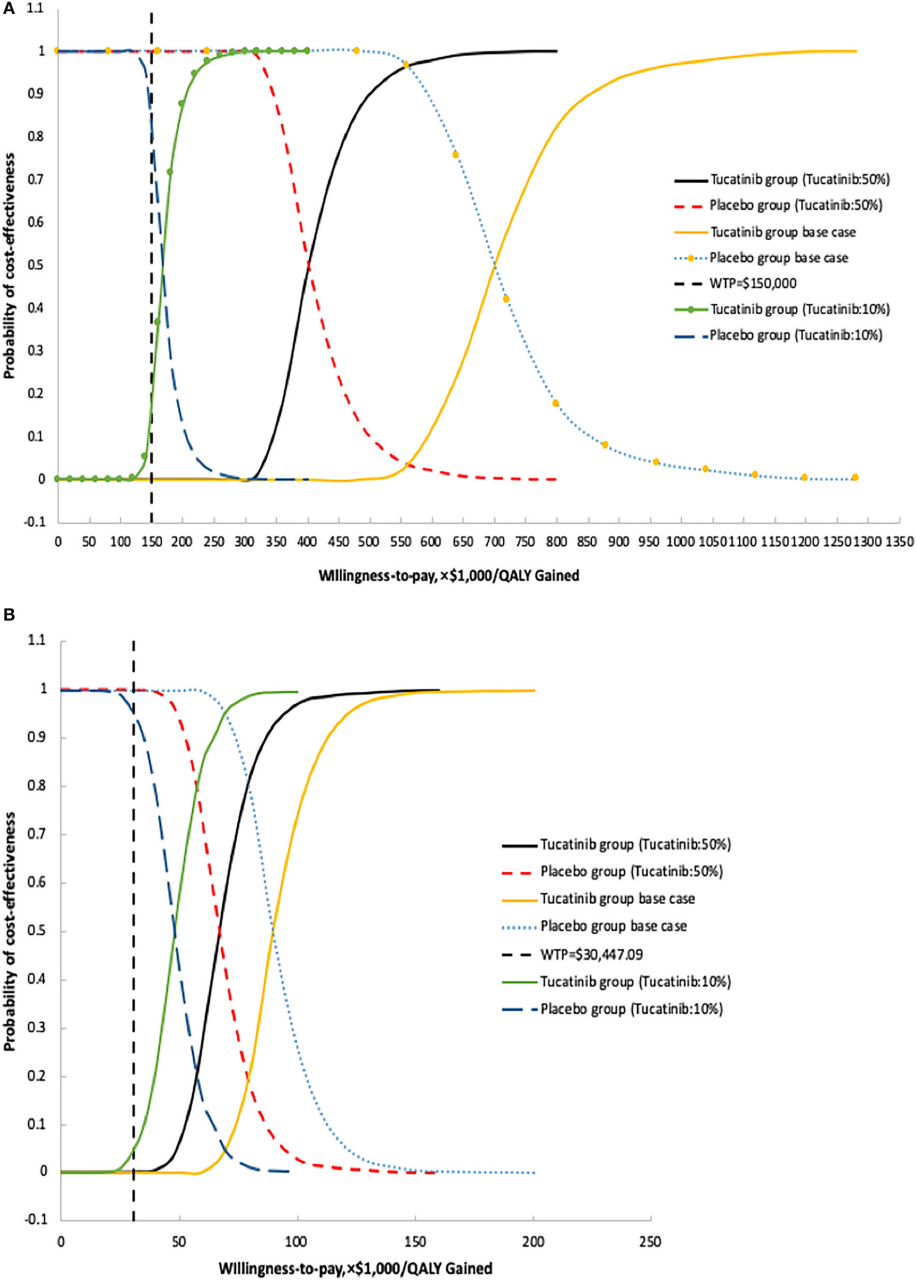
Probabilistic sensitivity analysis for cost effectiveness of treatment strategies for HER2 positive metastatic breast cancer. Cost-effectiveness acceptable curves are showing the cost-effective probability of tucatinib, trastuzumab, and capecitabine at different prices from the United States **(A)** and Chinese **(B)** perspectives. The dotted vertical lines represent the willingness to pay thresholds.

The results of the probabilistic sensitivity analysis show that, in the United States, when the WTP threshold adjusted to $500,000 or $800,000, the probability that TXT is cost-effective compared with TX is 0.1 and 80.3%, respectively (Table 2). For China, when the WTP threshold adjusted to $80,000 or $100,000, the probability of cost-effective in TXT group is 21.9 and 74.5%, respectively (Table 2). In the United States, when the WTP threshold is $150,000/QALY, and the unit price of tucatinib is 50 and 10% of the current price, the probability of cost-effective in the TXT group is 0 and 17.4%, respectively (Table 2, Figure 4). With a WTP threshold of $30,447.09 in China, the probability of a cost-effective in the TXT group is 0%. With the same discount ratio of tucatinib cost in China, the probabilities of cost-effective in the TXT group were 0 and 4.1%, respectively (Table 2, Figure 4).

## Discussion

To our knowledge, our study is the first cost effectiveness analysis of tucatinib, capecitabine plus trastuzumab compared with capecitabine plus trastuzumab for patients with Her-2 positive MBC from the United States and China. The ICER at base case estimate for TXT vs. TX was $699,976.43/QALY in the United States and $90,585.57/QALY in China, both of which were higher than the WTP threshold of $150,000/QALY and $30,447.09/QALY, respectively. This indicates that TXT is unlikely to be a cost-effective treatment for MBC. The current incidence of MBC in the United States is 7.2 per 100,000 population at risk (44), and 20–25% of patients overexpress HER2 (8); therefore, if TXT therapy were universally implemented at current prices, it would add $699 million per year to health care costs. For China, we estimated that the additional tucatinib treatment would increase the annual health burden by at least $724 million because of the large population base (8, 45, 46).

For patients with brain metastases, the increased cost of obtaining a QALY in the United States and China decreased to $395,373.81 and $56,403.31, respectively, which is mainly due to the more obvious difference of QALY in the brain metastasis group, suggesting the addition of tucatinib to capecitabine, and trastuzumab was most cost-effective for this subset. The survival benefit with tucatinib was observed in all subgroups tested of HER2CLIMB trial (15). Due to the lack of relevant data, we did not analyze subgroups other than brain metastases in our analysis. Therefore, from a more foresighted perspective, screening more appropriate patients will allow tucatinib treatment more likely to be cost-effective.

The univariable sensitivity showed that the parameter with the greatest influence on the ICER is the cost of tucatinib both in the United States and in China, which is consistent with the cost-effectiveness analysis of many innovative drugs in the treatment of advanced tumors (47–49). Probabilistic sensitivity analysis shows that if the price of tucatinib decreased by 90%, and the WTP threshold of China increases to $48,500, the probability of cost-effectiveness of adding tucatinib would be increased to 50%. Higher WTP may be achieved in some wealthy regions of China. According to statistics in 2018, there were four provincial-level administrative units in China (Beijing, Tianjin, Shanghai, and Jiangsu), whose three times GDP per capita were more than $48,500, involving about 140 million people (50). The hypothetical price reduction strategy may apply to these regions.

In January 2017, the State Council of China issued the National 13th 5 Year Plan to deepen the reform plan for the medical and health system, and many anti-cancer drugs price reduced after negotiations and were covered by medical insurance after entering the Chinese market. The medical insurance payment ratio of most anti-cancer drugs can reach about 70%. For example, the price of Pertuzumab after approval by the China Food and Drug Administration is 1,342.86 yuan per milliliter, but after negotiated price reduction and medical insurance reimbursement, patients only need to pay about 107.14 yuan per milliliter, and the cost has dropped by more than 90%. Changes in the actual cost of drugs provide tucatinib with the possibility to be cost-effective after entering China.

Tucatinib is not cost-effective for the United States at the current price, with the WTP threshold of $150,000/QALY. When the cost is reduced to $7.03/100 mg, the ICER of TXT compared to TX will be equal to the current WTP. Besides, the cost-effectiveness conclusion is the same between the two countries but with very different ICERs. Health care costs in China are far lower than in the United States, resulting in the ICER in the United States nearly eight times higher than in China. The limited transparency and absent federal control of drugs in American result in the highest drug costs worldwide (51). On May 11, 2018, the US administration released American Patients First to cut drug prices and decrease out-of-pocket payments (52). Significant price reduction or financial assistance is essential for patients to access innovative treatments and minimizing financial toxicity.

In fact, for patients with advanced cancer whose survival is limited, not only the TKIs, many anti-cancer drugs are not cost-effective due to their modest incremental benefit and high cost. Durkee et al. (53) published a cost of $472,668 per QALY for patients treated with first-line Pertuzumab for patients with MBC. Another study by Liao et al. (43) reported a cost of $300,564 per QALY for patients with fulvestrant plus anastrozole for hormone-receptor-positive MBC in post-menopausal women. Diaby et al. (54) reported a cost of $158,961.4 per QALY for patients treated with second-line for MBC. The use of innovative drugs that have been proven to be effective in clinical trials may lead to a substantial increase in medical expenditure, while the abandonment of these drugs means rejection of possible beneficial treatment (29). Therefore, cost-effectiveness analysis from different perspectives has become an important part of a broader discussion in how we allocate resources to treat cancer.

As in other cost-effectiveness analyses, our study has several limitations that are worth discussing. Firstly, the model input data come mainly from the results of the HER2CLIMB trial. For example, the Asian population accounts for only 4.4% of the HER2CLIMB trial (15), which may not accurately reflect the treatment effect of Chinese patients. Moreover, we did not have access to individual patients ' data, but survival data in our fitting curve were not significantly different from the results of the HER2CLIMB trial (Figure 2). Secondly, since the HER2CLIMB trial did not publish quality of life utility data, we assumed that the patients' quality of life was similar to that of previous studies (41–43). Also, we assumed the utility values of Chinese patients is equal to that of West. However, a range of ± 30% of utility values in the sensitivity analysis was used to analyze the effect of changes on the results. Thirdly, since tucatinib has not been marketed in China, the model's drug price is according to other drugs. We calculated the 50 and 90% off the model price of tucatinib, which will most likely include the lowest price value of tucatinib after approval. Fourth, we assumed in the model that all patients received the best supportive care after progression. This assumption is not completely consistent with clinical practice. However, patients in the HER2CLIMB trial have previously received trastuzumab, pertuzumab, and T-DM1 treatment, and 48% of these patients have brain metastases. The Nation Comprehensive Cancer Network guidelines and Chinese guidelines suggest the best supportive treatment as an option that may be taken into consideration for subsequent treatment (55, 56). Besides, the sensitivity analysis results show that the impact of subsequent treatment costs is considered limited.

In conclusion, from the payer's perspective in the United States and China, tucatinib is unlikely to be a cost-effective treatment for MBC at the current price. New pricing, screening the dominant patients, generic drugs, and new payment systems are needed to support cost-effective treatment measures, and our analysis provides valuable recommendations.

## Data Availability Statement

All datasets generated for this study are included in the article/supplementary material.

## Author Contributions

QW coordinated and performed data analyses, reported study results, and drafted the manuscript. WL and MZ assisted with the analyses. JH and PZ contributed to the interpretation of the results and reviewed and revised the manuscript. All authors read and approved the final manuscript.

## Conflict of Interest

The authors declare that the research was conducted in the absence of any commercial or financial relationships that could be construed as a potential conflict of interest.
